# Redetermination of the low-temperature polymorph of Li_2_MnSiO_4_ from single-crystal X-ray data

**DOI:** 10.1107/S1600536812035040

**Published:** 2012-08-15

**Authors:** Mineo Sato, Tadashi Ishigaki, Kazuyoshi Uematsu, Kenji Toda, Hirokazu Okawa

**Affiliations:** a Department of Chemistry and Chemical Engineering, Faculty of Engineering, Niigata University, Ikarashi 2-no-cho, Niigata City 950-2181, Japan; b Center for Transdiciplinary Research, Niigata University, 8050 Ikarashi 2-no-cho, Niigata 950-2181, Japan; c Graduate School of Science and Technology, Niigata University, 8050 Ikarashi 2-nocho, Niigata 950-2181, Japan; d Department of Earth Science and Technology, Faculty of Engineering and Resource Science, Akita University, Tegata Gakuen-machi, Akita 010-8502, Japan

## Abstract

Crystals of dilithium manganese(II) silicate were grown under high-temperature hydro­thermal conditions in the system LiOH—MnO_2_—SiO_2_. The title compound crystallizes in the β_II_-Li_3_PO_4_ structure type. The coordination polyhedra of all cations are slightly distorted tetra­hedra (*m* symmetry for MnO_4_ and SiO_4_), which are linked by corner-sharing to each other. The vertices of the tetra­hedra point to the same direction perpendicular to the distorted hexa­gonal close-packed (hcp) array of O atoms within which half of the tetra­hedral voids are occupied by cations. In comparison with the previous refinement from powder X-ray data [Dominko *et al.* (2006 ▶). *Electrochem. Commun.* **8**, 217–222], the present reinvestigation from single-crystal X-ray data allows a more precise determination of the distribution of the Li^+^ and Mn^2+^ cations, giving a perfectly site-ordered structure model for both Li^+^ and Mn^2+^.

## Related literature

For background to structural studies of Li_2_ *M*SiO_4_ (*M* = Mn, Fe, Co) compounds, see: Islam *et al.* (2011 ▶); Santamaría-Pérez *et al.* (2012 ▶); Setoguchi (1988 ▶); Yamaguchi *et al.* (1979 ▶). Polymorphism of Li_2_MnSiO_4_ was reported by Arroyo-de Dompablo *et al.* (2006 ▶, 2008 ▶); Belharouak *et al.* (2009 ▶); Dominko *et al.* (2006 ▶); Kokalj *et al.* (2007 ▶); Politaev *et al.* (2007 ▶); Wu *et al.* (2009 ▶); Zhong *et al.* (2010 ▶). For notation of Li_3_PO_4_ polymorphs, see: West & Glasser (1972 ▶). For theo­retical studies of the redox potentials and Li migration paths of Li_2_MnSiO_4_, see: Kuganathan & Islam (2009 ▶); Mali *et al.* (2010 ▶); Duncan *et al.* (2011 ▶), and for NMR studies of this material, see: Sirisopanaporn *et al.* (2011 ▶). For the bond-valence method, see: Brown & Altermatt (1985 ▶). For crystallographic background, see: Cooper *et al.* (2002 ▶).

## Experimental

### 

#### Crystal data

Li_2_MnSiO_4_
*M* *_r_* = 160.91
Orthorhombic, 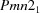

*a* = 6.3133 (16) Å
*b* = 5.3677 (14) Å
*c* = 4.9685 (12) Å
*V* = 168.37 (7) Å^3^
*Z* = 2
Mo *K*α radiation
μ = 4.11 mm^−1^
*T* = 295 K
0.26 × 0.19 × 0.18 mm

#### Data collection

Rigaku Mercury375R diffractometer
Absorption correction: multi-scan (*REQAB*; Rigaku, 1998 ▶) *T*_min_ = 0.377, *T*_max_ = 0.477
1636 measured reflections
423 independent reflections
419 reflections with *I* > 2σ(*I*)
*R*_int_ = 0.019

#### Refinement

*R*[*F* ^2^ > 2σ(*F* ^2^)] = 0.015
*wR*(*F* ^2^) = 0.037
*S* = 1.14
423 reflections
45 parameters
1 restraint
Δρ_max_ = 0.25 e Å^−3^
Δρ_min_ = −0.62 e Å^−3^
Absolute structure: Flack (1983 ▶), 189 Friedel pairs
Flack parameter: 0.171 (15)

### 

Data collection: *CrystalClear* (Rigaku, 2010 ▶); cell refinement: *CrystalClear*; data reduction: *CrystalClear*; program(s) used to solve structure: *SIR97* (Altomare *et al.*, 1999 ▶); program(s) used to refine structure: *SHELXL97* (Sheldrick, 2008 ▶); molecular graphics: *VESTA* (Momma & Izumi, 2011 ▶); software used to prepare material for publication: *WinGX* (Farrugia, 1999 ▶).

## Supplementary Material

Crystal structure: contains datablock(s) global, I. DOI: 10.1107/S1600536812035040/wm2658sup1.cif

Structure factors: contains datablock(s) I. DOI: 10.1107/S1600536812035040/wm2658Isup2.hkl

Additional supplementary materials:  crystallographic information; 3D view; checkCIF report

## Figures and Tables

**Table:** 

Li1—O1^i^	1.936 (10)
Li1—O3	1.956 (6)
Li1—O3^ii^	1.99 (2)
Li1—O2^iii^	2.009 (6)
Mn1—O3^iv^	2.0585 (16)
Mn1—O1	2.065 (2)
Mn1—O2	2.090 (2)
Si1—O1^v^	1.631 (3)
Si1—O3	1.6331 (17)
Si1—O2^vi^	1.639 (2)

**Table:** 

O1^i^—Li1—O3	112.0 (7)
O1^i^—Li1—O3^ii^	107.5 (5)
O3—Li1—O3^ii^	107.7 (7)
O1^i^—Li1—O2^iii^	108.6 (6)
O3—Li1—O2^iii^	113.9 (5)
O3^ii^—Li1—O2^iii^	106.8 (6)
O3^iv^—Mn1—O3	124.58 (8)
O3^iv^—Mn1—O1	105.74 (5)
O3^iv^—Mn1—O2	107.31 (6)
O1—Mn1—O2	104.54 (8)
O1^v^—Si1—O3	109.35 (10)
O3—Si1—O3^vii^	109.58 (13)
O1^v^—Si1—O2^vi^	108.23 (13)
O3—Si1—O2^vi^	110.16 (10)

Symmetry codes: (i) 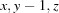; (ii) 
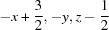
; (iii) 
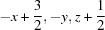
; (iv) 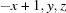; (v) 
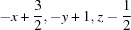
; (vi) 
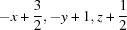
; (vii) 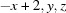.

**Table 2:** Bond-valence parameters derived from the present model and the previous studies.

Atom	Site	Present work	Dominko *et al.* ^1)^	Arroyo-de Dompablo *et al.* ^2)^
Li	4*b*	1.02 (6)	1.0 (1)	0.9
Mn	2*a*	1.89 (5)	2.1 (1)	1.77
Si	2*a*	3.89 (7)	3.6 (2)	3.65
O1	2*a*	2.02 (9)	1.9 (3)	1.75
O2	4*b*	1.97 (7)	1.9 (2)	1.86
O3	2*a*	1.87 (7)	2.0 (2)	1.90

1) The data, referred to Dominko *et al.* (2006 ▶), are based on the coordinates for primary *M*O_4_ (*M* = Li, Mn, Si) tetra­hedra. 2) The data, referred to Arroyo-de Dompablo *et al.* (2008 ▶), are based on the coordinates for primary *M*O_4_ (*M* = Li, Mn, Si) tetra­hedra optimized by density functional theory (DFT) methods.

**Fig. 1.**
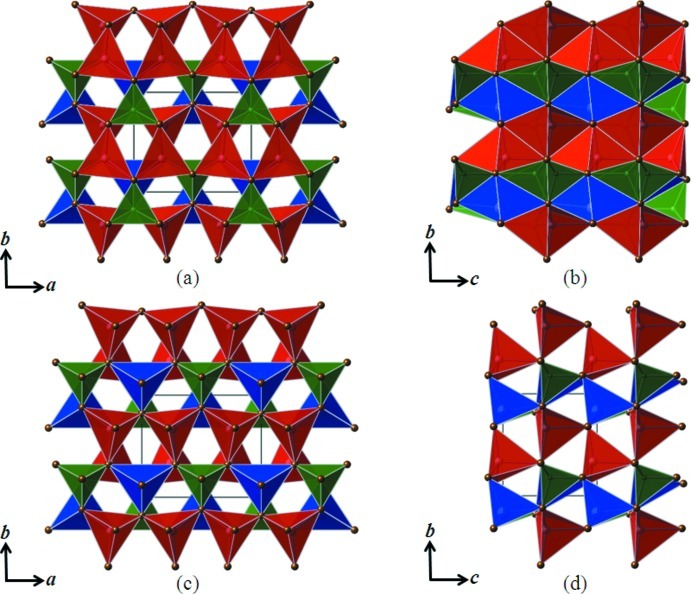
Structure model of Li2MnSiO4 determined by Dominko et al. (2006). The original structure model is projected (a) along [001] and (b) along [100], while the structure model where cation sites with low occupancies are removed is projected (c) along [001] and (d) along [100]. Polyhedra are indicated by red color for LiO4, blue color for MnO4, and green color for SiO4.

**Fig. 2.**
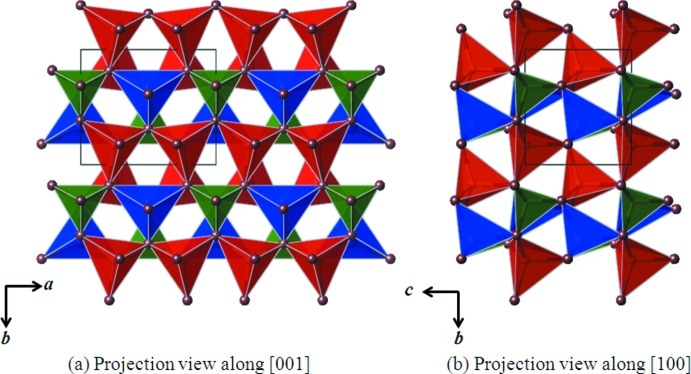
Structure model of Li2MnSiO4 determined by the present study with (a) projection view along [001] and (b) projection view along [100]. Polyhedra are indicated by red color for LiO4, blue color for MnO4, and green color for SiO4.

**Fig. 3.**
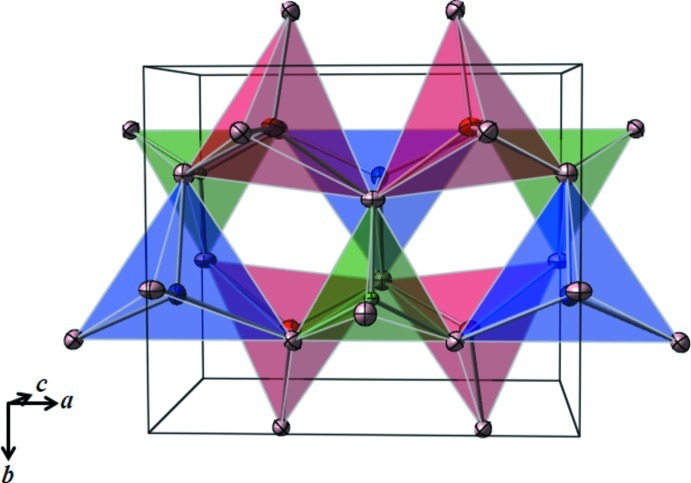
A perspective view of a part of the Li2MnSiO4 structure with the unit cell outlined. Displacement ellipsoids are drawn at the 70% probability level. Polyhedra are indicated by red color for LiO4, blue color for MnO4, and green color for SiO4.

## supplementary crystallographic information

### Comment

Lithium transition metal orthosilicates Li_2_*M*SiO_4_ (*M* = Mn, Fe, Co) have recently become attracting and received much attention as alternatives for the currently used cathode materials of lithium ion batteries, such as LiCoO_2_, Li(Ni,Mn,Co)O_2_, LiMn_2_O_4_ and Li*M*PO_4_ (*M* = Fe, Mn), because of the natural abundance of silica, iron, and manganese, but also due to a possible high theoretical capacity through two electron delivery. In order to understand the intercalation mechanism of Li^+^ ions in Li_2_*M*SiO_4_ cathode materials, their crystal structures have been investigated mainly by means of powder methods using X-ray or synchrotron radiation. Summarized by structural studies up-to-date (Islam *et al.*, 2011; Santamaría-Pérez *et al.*, 2012), it could be concluded that the Li_2_*M*SiO_4_ compounds (*M* = Fe, Mn, Co) belong to a large family of materials known as derivatives of Li_3_PO_4_, where oxygen atoms form arrays with a distorted hexagonal-close-packing (*hcp*) within which half of the tetrahedral voids are occupied by cations. Depending on which site (up or down) of the array the cations occupy, the material shows a rich polymorphism. Such compounds may be divided into two families, designated as *β*- and *γ*-forms after the notations used for Li_3_PO_4_ polymorphs (West & Glasser, 1972). The *γ*-polymorphs are built up of both corner- and edge-sharing tetrahedra with half of the tetrahedra pointing along one direction perpendicular to the *hcp* array and the other half pointing along the opposite direction, while the *β*-polymorphs are built up of only corner-sharing tetrahedra, with all the tetrahedra pointing to the same direction perpendicular to the *hcp* array. Detailed structural information, particularly for electrochemically active Li_2_MnSiO_4_, were available from the previous studies. Li_2_MnSiO_4_ exhibit three polymorphs, namely a low-temperature form denoted as *β*_II_ (*Pmn*2_1_), an intermediate temperature form denoted as *γ*_II_ (*Pmnb*), and a high-temperature form denoted as *γ*_o_ (*P*2_1_/*n*) (Arroyo-de Dompablo *et al.*, 2006, 2008; Belharouak *et al.*, 2009; Dominko *et al.*, 2006; Kokalj *et al.*, 2007; Politaev *et al.*, 2007; Wu *et al.*, 2009; Zhong *et al.*, 2010). It should be noted that in almost all polymorphs a site disorder for cationic sites, particularly for Li^+^ sites substituted by transition metal ions, was observed. Surprisingly, the structure models proposed for Li_2_*M*SiO_4_ (*M* = Mn, Fe, Co) have all been determined and refined by powder diffraction methods except for that of Li_2_CoSiO_4_ (Yamaguchi *et al.*, 1979). In terms of the fact that lithium has quite low scattering factors for X-rays, this may be true even for neutron diffraction, the crystallographic information obtained for Li sites by powder diffraction should inevitably include ambiguity to some extent. Efforts to obtain single crystals for structure determination have not been rewarded for Li_2_*M*SiO_4_ (*M* = Mn, Fe). Although Setoguchi (1988) succeeded to grow single crystals of Li_2_FeSiO_4_ from a flux method using LiCl at elevated temperatures, he could not determine the structure because of suffering from twinned crystals. Here we describe the single-crystal growth of Li_2_MnSiO_4_ by means of a high temperature hydrothermal method and its structure determination using single-crystal X-ray diffraction, confirming a perfectly site-ordered structure for Li_2_MnSiO_4_ in its low-temperature *β*_II_ (*Pmn*2_1_) polymorph.

The first detailed report on the description of the structure model for Li_2_MnSiO_4_ accompanied with numerical crystallographic data is probably that determined by Dominko *et al.* (2006) who performed Rietveld refinements in the space group *Pmn*2_1_ with *a* = 6.3109 (9) Å, *b* = 5.3800 (9) Å, *c* = 4.9662 (8) Å and *Z* = 2. The obtained structure model is presented in Fig. 1. It contains significant site disorders for all cationic sites, though the primary sites for the cations are located within the tetrahedral voids situated among a fairly distorted hexagonal-close-packing (*hcp*) of oxygen atoms. The *M*O_4_ (*M* = Li, Mn, Si) tetrahedra all point towards the same direction perpendicular to the *hcp* array, and are linked by corner-sharing. The partially occupied tetrahedral sites are located on the opposite sides of corresponding primary *M*O_4_ tetrahedra, the vertices of which point to the opposite direction. The central metal atom pairs are separated by distances of 1.0 (2) Å for Li—Li pairs, 0.52 (10) Å for Mn—Mn pairs and 1.09 (15) Å for Si—Si pairs, forming kinds of (pseudo) trigonal-bipyramidal *M*O_5_ polyhedra, as shown in Fig. 1(*b*). The structure can also be described as a typical *β*_II_-Li_3_PO_4_ structure if cation sites with low site-occupancies are removed, as shown in Fig. 1(*c*) and (*d*). This structure model, which has such excessive disorders, may have somewhat possible deficiencies. Furthermore, the environment around Li^+^ ions is crucially important for understanding the lithium intercalation behavior during the charge/discharge process. Theroretical studies concerned with expectation of redox potentials and lithium migration paths for Li_2_MnSiO_4_ cathodes have been accomplished by several groups (Kokalj *et al.* 2007; Kuganathan & Islam, 2009; Wu *et al.* 2009; Mali *et al.* 2010; Zhong *et al.* 2010; Duncan *et al.* 2011) based on the model by Dominko *et al.* (2006); most of these studies adopted the cation site disorder model or the idealized ordered one only with primary sites.

The structure refined in the present study is a perfectly site-ordered one for all cationic sites (Fig. 2) though the fundamental framework structure is the same as that previously reported. Notably, the displacement ellipsoids are relatively large not only for lithium atoms but also for manganese atoms (Fig. 3). This may reflect a high diffusibility both of Li^+^ and Mn^2+^ ions in this cathode material. The ordered structure model found in the present study is consistent with the results of an NMR study by Sirisopanaporn *et al.* (2011). Unexpectedly, information on atomic coordinates available for Li_2_MnSiO_4_ is scarce in the literature, where the data were refined from powder diffraction analyses (Dominko *et al.*, 2006) and obtained from an optimization by atomistic simulation (Arroyo-de Dompablo *et al.*, 2008). Table 2 shows the results of the bond-valence sum (BVS) analysis (Brown & Altermatt, 1985) for cation tetrahedra estimated from refined atomic coordinates in Li_2_MnSiO_4_, together with those for the structure models proposed previously for comparison. The deviations from the formal valences of each ion are fairly large for the previous studies, in particularly for Si, while in the present study the values of the BVS calculation for all ions are in very good agreement with the theoretical ones. Moreover, it should be mentioned that the *M*O_4_ tetrahedra in the present model have much more regular environments than the previous models.

No structural data based on single-crystal X-ray diffraction data have been reported for Li_2_MnSiO_4_, although recent works on positive electrode materials for rechargeable lithium batteries reported the electrochemical characterization of this cathode material. Our present structural study of Li_2_MnSiO_4_ provides more accurate information of its crystal structure than has been available up to now.

### Experimental

In order to synthesize Li_2_MnSiO_4_ single crystals, a high-temperature, high-pressure hydrothermal synthetic method was performed in a silver ampoule contained in a home-made autoclave made of stainless steel (SUS304) with 6 cm in outer diameter, 0.8 cm in inner diameter, and 1.8 cm^3^ in volume. The pressure was provided by water. High-purity chemical reagents of MnO_2_, SiO_2_ and LiOH^.^2H_2_O were used as reaction agents. A reaction mixture of LiOH, MnO_2_ and SiO_2_ with a molar ratio of Li:Mn:Si = 2:12:2 in a 3 cm long silver ampoule (inside diameter = 0.6 cm) was heated at 823 K for 3 days. The pressure was estimated to be 12 MPa at the reaction temperature according to the pressure-temperature diagram of pure water. The autoclave was then cooled to 323 K at 5 K/h and quenched to room temperature by removing the autoclave from the furnace. The product was filtered off, washed with water, rinsed with ethanol, and dried at ambient temperature. The reaction produced light-green rod-shaped crystals of Li_2_MnSiO_4_ that were obtained as a major product along with some quartz crystals.

The surface of the single crystals was observed by using optical (Olympus BX-60) and scanning electron microscopy (SEM, Jeol JSM-5310LVB). The elemental composition of the crystals was characterized by energy dispersive X-ray spectroscopy (EDS) attached to SEM (SEM/EDS, Nippon Denshi JED-2140).

### Refinement

The structure was solved by direct methods and refined by subsequent Fourier syntheses, leading to *wR*2 = 4.17% in the early stages of refinement. The relatively high value of the Flack parameter, *x* = 0.167, pointed to a possible twinned crystal. The examination using *ROTAX* (Cooper *et al.*, 2002) indicated two possible rotation twin axes about [010] and [001]. In addition to these rotation twin formations, a racemic twin (inversion twin) formation can be also possible for the non-centrosymmetric *Pmn*2_1_ space group. Subsequent refinements using the twin laws for the three cases yielded a satisfactory solution with *wR*2 = 3.67% for all cases and a Flack parameter *x* = 0.00 (2) for the rotation twin cases. The twin fraction ratio is 82.9: 17.1. In non-centrosymmetric space groups where mirror planes and/or glide planes exist, an inversion twin is equivalent to the rotation twin through the 2-fold rotation axis perpendicular to the mirror and/or the glide planes. This is true for the present case.

### Crystal data

**Table:**

Li_2_MnSiO_4_	*F*(000) = 154
*M**_r_* = 160.91	*D*_x_ = 3.174 Mg m^−^^3^
Orthorhombic, *P**m**n*2_1_	Mo *K*α radiation, λ = 0.71069 Å
Hall symbol: P 2ac -2	Cell parameters from 1684 reflections
*a* = 6.3133 (16) Å	θ = 3.2–27.5°
*b* = 5.3677 (14) Å	µ = 4.11 mm^−^^1^
*c* = 4.9685 (12) Å	*T* = 295 K
*V* = 168.37 (7) Å^3^	Prism, light green
*Z* = 2	0.26 × 0.19 × 0.18 mm

### Data collection

**Table:**

Rigaku Mercury375R diffractometer	423 independent reflections
Radiation source: Sealed Tube	419 reflections with *I* > 2σ(*I*)
Graphite monochromator	*R*_int_ = 0.019
Detector resolution: 13.6612 pixels mm^-1^	θ_max_ = 27.4°, θ_min_ = 3.8°
profile data from ω–scans	*h* = −8→8
Absorption correction: multi-scan (*REQAB*; Rigaku, 1998)	*k* = −6→6
*T*_min_ = 0.377, *T*_max_ = 0.477	*l* = −6→6
1636 measured reflections	

### Refinement

**Table:**

Refinement on *F*^2^	Secondary atom site location: difference Fourier map
Least-squares matrix: full	*w* = 1/[σ^2^(*F*_o_^2^) + (0.0217*P*)^2^] where *P* = (*F*_o_^2^ + 2*F*_c_^2^)/3
*R*[*F*^2^ > 2σ(*F*^2^)] = 0.015	(Δ/σ)_max_ < 0.001
*wR*(*F*^2^) = 0.037	Δρ_max_ = 0.25 e Å^−^^3^
*S* = 1.14	Δρ_min_ = −0.62 e Å^−^^3^
423 reflections	Extinction correction: *SHELXL97* (Sheldrick, 2008), Fc^*^=kFc[1+0.001xFc^2^λ^3^/sin(2θ)]^-1/4^
45 parameters	Extinction coefficient: 0.392 (13)
1 restraint	Absolute structure: Flack (1983), 189 Friedel pairs
0 constraints	Flack parameter: 0.171 (15)
Primary atom site location: structure-invariant direct methods	

### Special details

**Table:**

Geometry. All s.u.'s (except the s.u. in the dihedral angle between two l.s. planes) are estimated using the full covariance matrix. The cell s.u.'s are taken into account individually in the estimation of s.u.'s in distances, angles and torsion angles; correlations between s.u.'s in cell parameters are only used when they are defined by crystal symmetry. An approximate (isotropic) treatment of cell s.u.'s is used for estimating s.u.'s involving l.s. planes.
Refinement. Refinement of *F*^2^ against ALL reflections. The weighted *R*-factor *wR* and goodness of fit *S* are based on *F*^2^, conventional *R*-factors *R* are based on *F*, with *F* set to zero for negative *F*^2^. The threshold expression of *F*^2^ > 2*σ*(*F*^2^) is used only for calculating *R*-factors(gt) *etc*. and is not relevant to the choice of reflections for refinement. *R*-factors based on *F*^2^ are statistically about twice as large as those based on *F*, and *R*- factors based on ALL data will be even larger.

### Fractional atomic coordinates and isotropic or equivalent isotropic displacement parameters (Å^2^)

**Table:**

	*x*	*y*	*z*	*U*_iso_*/*U*_eq_	
Li1	0.7503 (4)	−0.1688 (4)	0.995 (5)	0.0090 (7)	
Mn1	0.5	0.33172 (5)	0.9968	0.00733 (15)	
Si1	1	0.32090 (9)	0.9851 (3)	0.00350 (17)	
O1	0.5	0.6868 (3)	1.1569 (5)	0.0071 (5)	
O2	0.5	0.3867 (3)	0.5804 (4)	0.0081 (4)	
O3	0.7887 (2)	0.1799 (2)	1.0979 (4)	0.0075 (3)	

### Atomic displacement parameters (Å^2^)

**Table:**

	*U*^11^	*U*^22^	*U*^33^	*U*^12^	*U*^13^	*U*^23^
Li1	0.0084 (17)	0.0061 (15)	0.0125 (15)	−0.0009 (7)	0.0000 (14)	−0.002 (2)
Mn1	0.0055 (2)	0.0072 (2)	0.0093 (2)	0	0	0.0000 (2)
Si1	0.0035 (3)	0.0028 (3)	0.0042 (4)	0	0	−0.0003 (3)
O1	0.0075 (9)	0.0083 (8)	0.0056 (11)	0	0	−0.0004 (6)
O2	0.0098 (8)	0.0048 (7)	0.0096 (10)	0	0	0.0001 (7)
O3	0.0068 (5)	0.0067 (6)	0.0089 (7)	−0.0009 (4)	0.0008 (7)	0.0000 (5)

### Geometric parameters (Å, º)

**Table:**

Li1—O1^i^	1.936 (10)	Mn1—O1	2.065 (2)
Li1—O3	1.956 (6)	Mn1—O2	2.090 (2)
Li1—O3^ii^	1.99 (2)	Si1—O1^v^	1.631 (3)
Li1—O2^iii^	2.009 (6)	Si1—O3	1.6331 (17)
Mn1—O3^iv^	2.0585 (16)	Si1—O3^vi^	1.6331 (17)
Mn1—O3	2.0585 (15)	Si1—O2^vii^	1.639 (2)
			
O1^i^—Li1—O3	112.0 (7)	O3^iv^—Mn1—O2	107.31 (6)
O1^i^—Li1—O3^ii^	107.5 (5)	O3—Mn1—O2	107.31 (6)
O3—Li1—O3^ii^	107.7 (7)	O1—Mn1—O2	104.54 (8)
O1^i^—Li1—O2^iii^	108.6 (6)	O1^v^—Si1—O3	109.35 (10)
O3—Li1—O2^iii^	113.9 (5)	O1^v^—Si1—O3^vi^	109.35 (10)
O3^ii^—Li1—O2^iii^	106.8 (6)	O3—Si1—O3^vi^	109.58 (13)
O3^iv^—Mn1—O3	124.58 (8)	O1^v^—Si1—O2^vii^	108.23 (13)
O3^iv^—Mn1—O1	105.74 (5)	O3—Si1—O2^vii^	110.16 (10)
O3—Mn1—O1	105.74 (5)	O3^vi^—Si1—O2^vii^	110.16 (10)

Symmetry codes: (i) *x*, *y*−1, *z*; (ii) −*x*+3/2, −*y*, *z*−1/2; (iii) −*x*+3/2, −*y*, *z*+1/2; (iv) −*x*+1, *y*, *z*; (v) −*x*+3/2, −*y*+1, *z*−1/2; (vi) −*x*+2, *y*, *z*; (vii) −*x*+3/2, −*y*+1, *z*+1/2.

### Bond-valence parameters derived from the present model and the previous studies.

**Table:**

Atom	Site	Present work	Dominko *et al.*^1)^	Arroyo-de Dompablo *et al.*^2)^
Li	4*b*	1.02 (6)	1.0 (1)	0.9
Mn	2*a*	1.89 (5)	2.1 (1)	1.77
Si	2*a*	3.89 (7)	3.6 (2)	3.65
O1	2*a*	2.02 (9)	1.9 (3)	1.75
O2	4*b*	1.97 (7)	1.9 (2)	1.86
O3	2*a*	1.87 (7)	2.0 (2)	1.90

1) The data, referred to Dominko *et al.* (2006), are based on the coordinates for primary *M*O_4_ (*M* = Li, Mn, Si) tetrahedra. 2) The data, referred to Arroyo-de Dompablo *et al.* (2008), are based on the coordinates for primary *M*O_4_ (*M* = Li, Mn, Si) tetrahedra optimized by density functional theory (DFT) methods.
